# Purification and Application of Genetically Encoded Potassium Ion Indicators for Quantification of Potassium Ion Concentrations within Biological Samples

**DOI:** 10.1002/cpch.71

**Published:** 2019-09

**Authors:** H. Bischof, S. Burgstaller, N. Vujic, T. Madl, D. Kratky, W. F. Graier, R. Malli

**Affiliations:** 1 Gottfried Schatz Research Center, Molecular Biology and Biochemistry, Medical University of Graz, Graz, Austria; 2 BioTechMed-Graz, Graz, Austria

**Keywords:** cell viability, FRET, GEPIIs, K^+^, potassium ions, recombinant protein, serum K^+^

## Abstract

Vital cells maintain a steep potassium ion (K^+^) gradient across the plasma membrane. Intracellular potassium ion concentrations ([K^+^]) and especially the [K^+^] within the extracellular matrix are strictly regulated, the latter within a narrow range of ~3.5 to 5.0 mM. Alterations of the extracellular K^+^ homeostasis are associated with severe pathological alterations and systemic diseases including hypo- or hypertension, heart rate alterations, heart failure, neuronal damage or abnormal skeleton muscle function. In higher eukaryotic organisms, the maintenance of the extracellular [K^+^] is mainly achieved by the kidney, responsible for K^+^ excretion and reabsorption. Thus, renal dysfunctions are typically associated with alterations in serum- or plasma [K^+^]. Generally, [K^+^] quantifications within bodily fluids are performed using ion selective electrodes. However, tracking such alterations in experimental models such as mice features several difficulties, mainly due to the small blood volume of these animals, hampering the repetitive collection of sample volumes required for measurements using ion selective electrodes. We have recently developed highly sensitive, genetically encoded potassium ion indicators, the GEPIIs, applicable for in vitro determinations of [K^+^]. In addition to the determination of [K^+^] within bodily fluids, GEPIIs proved suitable for the real-time visualization of cell viability over time and the exact determination of the number of dead cells.

## Introduction

Potassium ions (K^+^) are fundamentally involved in multiple cellular and systemic functions (Ceccarelli, Fesce, Grohovaz, & Haimann, 1988; Larkin, Brown, Goldstein, & Anderson, 1983; Page & Di Cera, 2006; Toda, 1969). The maintenance of extracellular K^+^ concentrations ([K^+^]_ex_) is especially essential and, thus, is tightly regulated in a range between 3.5 and 5.0 mM in humans (Thier, 1986). Consequently, it is not surprising that disturbances in K^+^ homeostasis are associated with severe pathological alterations, including hypo- or hypertension, heart rate alterations, heart failure, neuronal damage, or skeletal muscle dysfunction (Antunes et al., 2014; Entz et al., 2016; Haddy, Vanhoutte, & Feletou, 2006; Kardalas et al., 2018; Lehnhardt & Kemper, 2011; Sica et al., 2002). Typically, the measurement of K^+^ concentrations within human serum or plasma samples represents a standard clinical procedure determined by ion-selective electrodes (ISEs; Rastegar, 1990). Such measurements allow tracking and tightly controlling [K^+^]_ex_ in individuals, and often serve as an indicator of renal damage (Kunis & Charney, 1981). However, [K^+^] measurements using ISEs generally require sample volumes of several milliliters. Although this is not critical in humans, such sample volumes become a critical parameter when working with small laboratory animals such as mice, which possess very small blood volumes of less than 2 ml (Riches, Sharp, Thomas, & Smith, 1973). To develop pharmacologically active substances that might have an impact on blood K^+^ levels in mammals, tracking extracellular K^+^ dynamics in small laboratory animals is inevitable and requires alternative applicable approaches for K^+^ quantifications.

In addition to [K^+^]_ex_, the intracellular [K^+^] ([K^+^]_in_) is also very strictly controlled (Checchetto, Teardo, Carraretto, Leanza, & Szabo, 2016; Palmer, 2015). Vital cells maintain a steep K^+^ gradient across the plasma membrane in order to keep the electrochemical gradient, which is important for numerous cellular functions (Ceccarelli et al., 1988; Larkin et al., 1983; Page & Di Cera, 2006). It has recently been demonstrated that [K^+^]_ex_ within the tumor microenvironment is elevated due to K^+^ release from necrotic cells (Eil et al., 2016). The measurement of [K^+^]_ex_ might represent a valuable alternative to available cell viability assays, allowing the visualization of cell death with high spatial and temporal resolution in vitro or in vivo. Here, we show that quantification of [K^+^]_ex_ in the supernatant of cultured cells allows time-resolved visualization of cell death.

**Figure 1:**
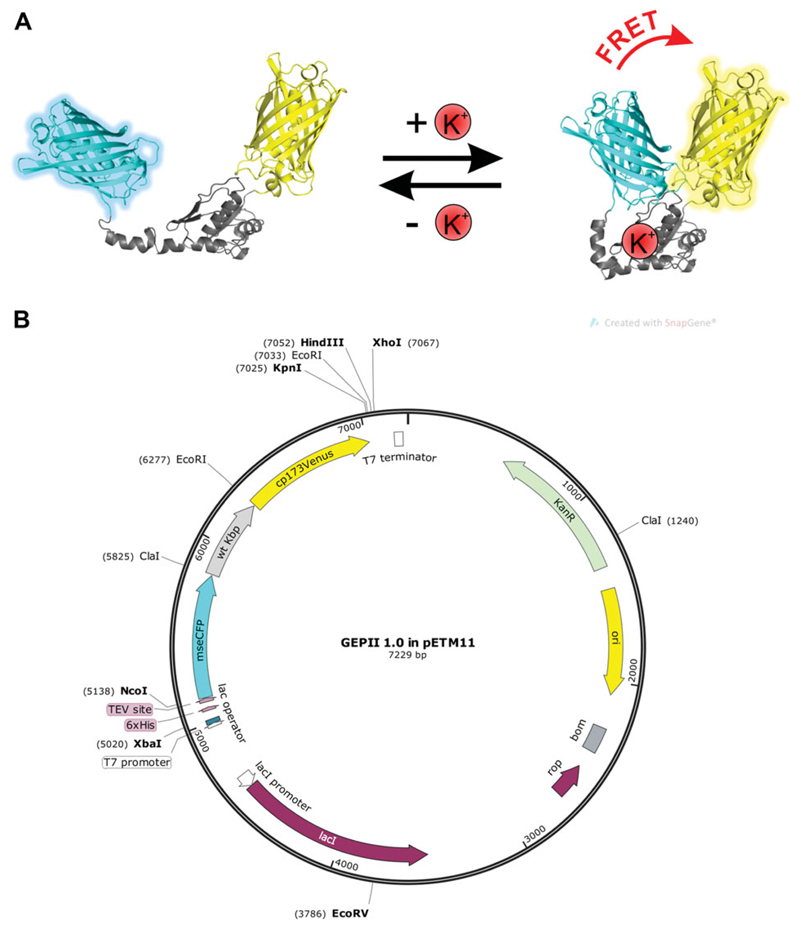
Functional principle and plasmid map of GEPII 1.0. (**A**) Schematic representation of the K^+^-sensing mechanism of GEPII 1.0. mseCFP (cyan), wild-type Kbp (grey), and circularly permuted Venus (yellow) are shown. (**B**) Plasmid map of GEPII 1.0 subcloned into pETM11 vector for bacterial expression. mseCFP (cyan), wild-type Kbp (wt Kbp, grey), and circularly permuted Venus (cp173Vens, yellow) as well as the most important features of the plasmid are indicated in the map. Single-cutting restriction enzymes (*Eco*RV, *Hin*dIII, *Kpn*I *Nco*I, *Xba*I, and *Xho*I, all in bold) as well as internal restriction sites with multiple cutting sites (*Cla*I and *Eco*RI) are indicated. Locations of the 6× His-Tag and a TEV protease site located between the His-Tag and mseCFP are shown.

We have recently developed a highly sensitive Förster resonance energy transfer (FRET)–based, genetically encoded K^+^ indicator referred to as GEPII 1.0 (Fig. 1A; Bischof et al., 2017). Subcloning of the GEPII 1.0 coding sequence into a vector for bacterial expression (pETM11; Fig. 1B) allowed purification of the recombinant protein. The use of the recombinant purified protein enables quantification of [K^+^] within various biological samples. Our data highlight the suitability of GEPIIs for repetitive [K^+^] measurements within samples of small laboratory animals that are as precise as the gold-standard instrument, the ISE, and use only a small drop of blood from the animal. In addition, the probe proved suitable for online visualization of cell viability within the supernatant of mammalian cells (Bischof et al., 2017).

Basic Protocol 1 describes the transformation, expression, and purification of recombinant GEPII 1.0 from *Escherichia coli*, yielding functional K^+^ probes in a K^+^-free solution. Basic Protocol 2 deals with the collection of murine blood samples, the preparation of serum from the blood, and the application of recombinant purified GEPII 1.0 for quantification of [K^+^] within tiny volumes of these samples. Basic Protocol 3 describes the use of the recombinant purified protein for online visualization of cell viability and growth. Finally, Basic Protocol 4 demonstrates how to estimate the number of dead cells using GEPII 1.0.

## Basic Protocol 1: Expression and Purification of Recombinant GEPII 1.0

The following protocol describes transformation of the plasmid encoding GEPII 1.0 into chemically competent *E. coli* cells and subsequent expression and purification of GEPII 1.0. Purification according to this protocol will yield 3-5 mg of highly pure and functional GEPII 1.0, suitable for quantification of [K^+^] in various biological samples.

### Materials

Chemically competent BL21(DE3) *E. coli* cells (New England Biolabs, cat. no. C2527I)
Plasmid encoding GEPII 1.0 for bacterial expression (Bac. GEPII 1.0, NGFI)
SOC medium (see recipe)
LB medium (see recipe) with 50 μg/ml kanamycin sulfate (Carl Roth, cat. no. T832.2)
50% glycerol solution (see recipe)
Isopropyl β-d-1-thiogalactopyranoside (IPTG; Sigma-Aldrich, cat. no. I6758.5G)
Lysis buffer (see recipe)
Benzonase nuclease (Sigma-Aldrich, cat. no. E1014)
Bacterial protease inhibitor cocktail (Carl Roth, cat. no. 3758.1)
Column storage buffer (see recipe)
Wash buffer 1 (see recipe)
Wash buffer 2 (see recipe)
High-salt purification buffer (see recipe)
Liquid nitrogen
SEC buffer (see recipe)
Dilution buffer (see recipe)
K^+^ calibration solutions (see recipe)

1.5-ml microcentrifuge tubes (Thermo Fisher Scientific, cat. no. 11926955)
Shaking incubator (Thermo Fisher Scientific, cat. no. SHKE8000-8CE)
ThermoStat Plus (Eppendorf, cat. no. 5360000011)
15-ml conical tubes (Eppendorf, cat. no. 00301222151)
Sorvall LYNX 6000 Superspeed centrifuge (Thermo Fisher Scientific, cat. no. 75006590) or equivalent
Sonicator (e.g., Ultrasonic Processor Q500, Qsonica, cat. no. Q500.110)
Econo-Pac Chromatography columns (Bio-Rad, cat. no. 7321010EDU)
Protino Ni-NTA agarose (Macherey-Nagel, cat. no. 745400.100)
Vivaspin 15R concentrator tubes (Sartorius, cat. no. VS15RH21)
Eppendorf centrifuge 5810 R (Eppendorf, cat. no. 5811000325)
Superdex 200 Increase 10/300 GL size-exclusion columns (GE Healthcare, cat. no. 28990944)
Äkta pure protein purification system (GE Healthcare, cat. no. 29018224)
Black, F-bottom 96-well plates (Greiner Bio-One, cat. no. 655086)
Fluorescence Plate Reader (CLARIOstar Plus, BMG Labtech)

Additional reagents and equipment for determining protein concentration

### Transform competent *E. coli*

*NOTE:* All following steps should be performed in as sterile an environment as possible. Thus, working in front of a Bunsen burner is necessary.

1. Let *E. coli* BL21(DE3) cells thaw on ice and aliquot 50 μl into a 1.5-ml microcentrifuge tube. Quickly re-freeze the tube containing the bacterial stock at −80°C.
2. Carefully add 50-100 ng plasmid DNA encoding GEPII 1.0 to the 50 μl of bacteria. Mix by carefully moving the pipette tip.
  *Do not vortex and do not blow out the remaining liquid from the pipette tip, as this may damage the cells*.
3. Incubate on ice for 30 min.
4. Heat shock the cells at 42°C for 10 s.
  *The time needed for heat shock may depend on the strain and competence of the bacteria*.
5. Incubate in ice for 5 min.
6. Add 950 μl SOC medium and incubate at 37°C for 1 h with shaking.
7. Add 10 ml LB medium containing 50 μg/μl kanamycin sulfate to 15-ml conical tubes. Inoculate with bacterial culture and cultivate overnight at 37°C.
8. Pipet 350 μl of 50% glycerol solution into sterile 1.5-ml microcentrifuge tubes and add 500 μl bacterial culture. Immediately freeze glycerol stocks at −80°C.
  *Prepared glycerol stocks can be used in the future to start directly with induction*.

### Induce protein expression

*NOTE*: From this point on, avoid excessive light exposure at all steps to prevent photo-bleaching of the fluorescent proteins.

9. Inoculate 1 L LB medium (with kanamycin) with transformed bacterial culture and incubate with shaking at 37°C until the OD_600_ reaches 0.6-0.8 (typically, 4-6 hr).
10. Add 1 mM IPTG and incubate for 4-6 hr at 20°C with shaking to induce protein expression.
  *After 4-6 hr, bacteria should have a slightly green to yellow color caused by expression of GEPII 1.0*.
11. Transfer bacterial suspension to centrifuge tubes and pellet cells at 7,800 × *g* (6,000 rpm in Sorvall LYNX Superspeed centrifuge) for 10 min at 4°C.
12. Remove supernatant and resuspend pelleted cells in 15 ml lysis buffer.
  *If desired, resuspended cells can be frozen at* −*80°C. Frozen cells should be thawed in a 37°C water bath before use*.

### Prepare cell lysate

13. Add 250 U Benzonase nuclease and 150 μl bacterial protease inhibitor cocktail (1:100 dilution) to the cell suspension and incubate on ice for 30 min.
14. Break up cells by sonicating for 20 min on ice. Perform 10 s of sonication followed by 10 s of pause over a period of 20 min.
  *Avoid touching the tube with the sonicator or the tube will be damaged and the probe lost. Keep suspension on ice to prevent damage from heat generated during sonication*.
15. Centrifuge at 17,200 × *g* (10,000 rpm in Sorvall LYNX) for 45 min at 4°C to pellet cell debris.
16. Transfer supernatant to 15-ml conical tubes and keep on ice. Discard pelleted debris.
  *If desired, centrifuged supernatant can be frozen at* −*80°C. Frozen supernatant should be thawed in a 37°C water bath before use*.

### Perform nickel affinity chromatography

17. Load an Econo-Pac Chromatography column with 3 ml pre-charged Protino Ni-NTA agarose.
  *Nickel-agarose should never be allowed to dry out. Columns can be stored in column storage buffer*.
18. Equilibrate column by filling to the top with lysis buffer (15 ml) and letting the buffer flow through.
  *If the next step cannot be performed immediately after equilibration, re-cap the column with a remaining volume of 2 ml lysis buffer on the beads or refill it to the top to prevent drying*.
19. Apply cell lysate to the column by carefully decanting or pipetting it.
  *Avoid disturbing the beads, as this may reduce the efficiency of protein binding*.
20. Allow the lysate to flow through the column.
  *The blue nickel beads may change color upon protein binding*.
  *The maximum flow rate should be 1 ml/min. If the flow rate is too high, it may be necessary to re-apply the flowthrough to the column to ensure optimal binding of the protein*.
21. Wash column once each with 15 ml wash buffer 1 and then 15 ml wash buffer 2.
  *The pH of both buffers must be adjusted to 8.0, as lower pH values may lead to elution of the protein*.
22. Elute protein with 15 ml of high-salt purification buffer.
  *If desired, the protein solution can be frozen in liquid nitrogen and further steps can be performed on the next day. Frozen eluate should be quickly thawed in a 37°C water bath before use*.
23. Concentrate the protein solution using Vivaspin concentrator tubes according to manufacturer’s instructions. Centrifuge at 2,300 × *g* (4,000 rpm in Eppendorf centrifuge) for 20 min at 4°C. Repeat centrifugation until a volume of 500 μl protein remains in the collector.
  *Discard the flowthrough after each centrifugation if there is too much liquid at the bottom of the tube*.
  *The protein is now concentrated enough to be applied to the size-exclusion column*.

### Perform size-exclusion chromatography

*NOTE:* Other FPLC systems may be used, but the protocol may vary depending on the system.

24. Insert a Superdex 200 increase 10/300 GL column into the Äkta pure system according to manufacturer’s instructions.
25. Choose the “inlet” and “column position” and define the “system flow”. Set the column position at “Bypass” to avoid applying any impurities onto the column during washing.
  *Inlet defines the tube that is connected to the equilibration buffer (SEC buffer). System flow is set to 0.5 ml/min*.
26. Wash the selected pump system with SEC buffer.
27. Under “monitors”, select “wavelengths” to define the wavelengths that are shown on the screen. Set wavelength to 480 nm to screen for the yellow fluorescent GEPII 1.0 protein.
28. Set “alarms” according to the used column. Define the pre-column pressure and the delta column pressure.
29. Press “execute” to wash the system without the column.
30. Select the “manual load” option on the injection loop and apply 5 ml SEC buffer with a syringe onto the loop.
31. Switch from “manual load” to “inject” to wash the loop.
  *Every loop has a defined volume that is retained in the loop. Any excess volume will flow into the waste. For 500 μl protein solution, use a 1-ml loop to ensure that the protein is not lost during application*.
32. Change back to “manual load” on the loop and then remove the syringe.
  *Do not remove the syringe without changing back to “manual load” or air will be trapped in the system*.
33. Change from the “bypass” position to the actual column position and allow 30 ml SEC buffer to flow through the column to wash and equilibrate it.
  *All solutions and buffers will now pass through the column*.
34. With “manual load” selected in the software, use a small syringe to load protein solution from the concentrator tubes onto the loop. Be careful to avoid any air bubbles.
35. Change to “inject” to apply the protein onto the column. After ~6 ml, start the fractionation to collect fractions containing protein.
36. Combine fractions containing protein and determine the protein concentration using a method of choice.
37. Calculate the molarity of the protein solution.
  *GEPII 1.0 has a size of* ~*70 kDa*.

### Verify functionality of purified GEPII 1.0

**Figure 2:**
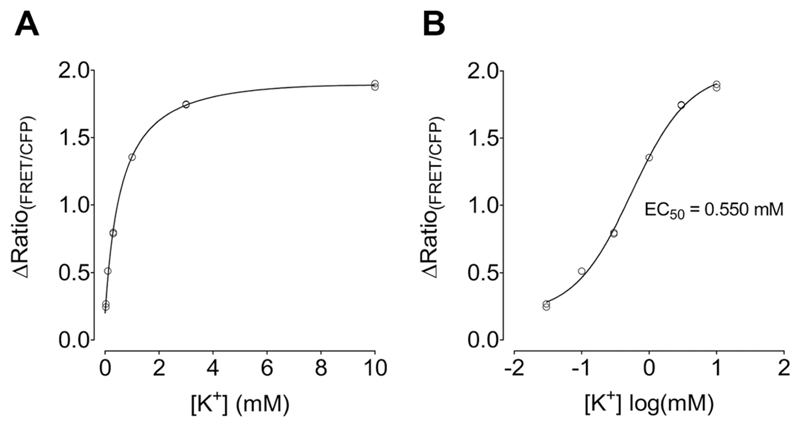
Testing the functionality of recombinant purified GEPII 1.0. Blank-corrected FRET ratio signals (∆Ratio_(FRET/CFP)_) of GEPII 1.0 are plotted against [K^+^], both linearly (**A**) and logarithmically (**B**), in mM. The EC_50_ of GEPII 1.0 determined by sigmoidal concentration-response curve fitting is indicated in (B). *n* = 2 experiments.

38. Dilute GEPII 1.0 in dilution buffer to a concentration of 400 nM and then pipet 40 μl into 14 wells of a black 96-well plate.
39. Add 40 μl of the following K^+^ calibration solutions to duplicate wells: 60 μM, 200 μM, 600 μM, 2 mM, 6 mM, and 20 mM. Add 40 μl dilution buffer to the last two wells as a blank.
  *The final [K*^+^*] in the wells will be 0 μM, 30 μM, 100 μM, 300 μM, 1 mM, 3 mM, and 10 mM*.
40. Measure all wells using the CLARIOstar Plus fluorescence plate reader with the following settings:
  Excitation: 430-20 nm
    Emission: 475-10 and 525-10 nm
    Dichroic filters: 455 and 480 nm
    Gain: 2,000 for both emissions with focal height adjusted to blank
    Top optics
41. Calculate FRET ratio values of all wells by determining the FRET/CFP fluorescence (525 nm/475 nm).
42. Determine the average FRET ratio value from the blank wells and subtract it from the value for each well containing K^+^ calibration solution. Plot the blank-corrected FRET/CFP ratios (∆Ratio) against [K^+^] both linearly and logarithmically and determine the EC_50_.
  *If GEPII 1.0 is functional, a concentration-dependent increase in the FRET ratio should be observed, with an EC_50_ of* ~*500-600 μM at room temperature, as shown in Figure 2*.
43. If GEPII 1.0 has proven functional, prepare aliquots as desired and shock-freeze in liquid nitrogen. Store up to 2 years at −80°C in the dark. If protein has been stored for >1 year, it is recommended to repeat the functionality tests (steps 38-42) before use.

## Basic Protocol 2: Collection of Murine Serum Samples and Determination of Serum K^+^ Concentration using GEPII 1.0

The following protocol describes how to collect murine blood by phlebotomy from v. facialis and how to prepare murine serum samples. Furthermore, presents the generation of a K^+^ calibration curve for GEPII 1.0 and the determination of [K^+^] in murine serum samples using GEPII 1.0.

Generally, mice (e.g., wild-type C57BL/6) should be maintained in a clean environment with a regular light-dark cycle (12 hr/12 hr) and unrestricted access to food and water. The mouse strain as well as conditions can be modified as needed to suit the experimental design.

### Materials

Mice
  Dilution buffer (see recipe)
  Serum assay solution (see recipe)
  K^+^ calibration solutions (see recipe)
  K^+^ standard solutions (see recipe)

Mediware blood lancets (Servoprax, cat. no. 10947551)
  1.5-ml microcentrifuge tubes (Thermo Fisher Scientific, cat. no. 11926955)
  Sterile gauze
  Microcentrifuge (Himac CT15RE, Koki Holdings, cat.no 90560701)
  Black, F-bottom 96-well plates (Greiner Bio-One, cat. no. 655086)
  Fluorescence plate reader (CLARIOstar Plus, BMG Labtech)

### Collect blood and prepare serum samples

1. On the day of sample collection, remove mouse from its environment and restrain it securely by grasping the neck scruff with the thumb and index finger and holding the base of the tail between the palm and the ring finger.
2. Puncture the superficial temporal vein using a sterile single-use lancet.
3. Collect blood from the second drop (~20-30 μl) into a 1.5-ml microcentrifuge tube.
4. Lightly press the puncture site with a sterile gauze for a few seconds to cause hemostasis. Release mouse from restraint and return to its home cage.
5. Repeat for the desired number of mice.
6. Allow blood samples to clot for 15 min at room temperature.
7. Centrifuge samples for 10 min at 400 × *g* to separate the blood clot from the serum. Carefully transfer serum to a new 1.5-ml microcentrifuge tube, taking care not to disturb the pelleted blood clot, and place on ice.
  IMPORTANT: *If the blood clot is mixed with the serum, start over with a fresh blood sample*.
8. Visually check sera for hemolysis.
  *If samples are discolored red, hemolysis may have occurred. If hemolysis is visible, be careful with these samples, as the lysis of red blood cells drastically increases the [K*^+^*]. When in doubt, exclude these samples and start over with fresh blood samples*.
  *Serum samples are ready for analysis and can be can be analyzed directly or stored for up to 1 year at* −*20°C. For direct analysis, samples should be maintained on ice*.

### Generate standard curve

**Figure 3:**
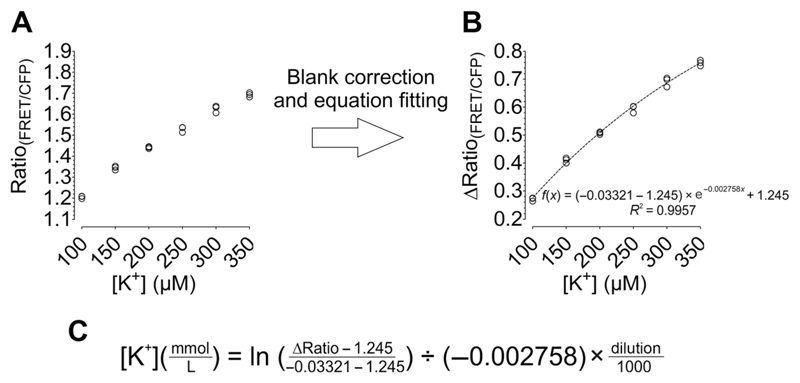
Generation of a calibration curve suitable for quantification of [K^+^] in serum samples. (**A**) FRET ratio signals of GEPII 1.0 are plotted against [K^+^] (*n* = 3) before (**A**) and after (**B**) blank correction. Data were fitted using a one-phase decay. The equation for the curve and *R*^2^ are indicated in (B). (**C**) Formula for calculation of [K^+^] (mM) in serum samples from FRET ratio signals of GEPII 1.0, determined by solving the equation in (B).

9. Pipet 40 μl serum assay solution into the number of wells of a 96-well plate needed to generate the calibration curve.
  *To generate the calibration curve, we recommend using at least triplicate wells for each concentration. Thus, if the calibration curve consists of a blank and six K*^+^ *concentrations, 21 wells are required to generate the calibration curve*.
10. Add 40 μl of each K^+^ calibration solution (200 μM, 300 μM, 400 μM, 500 μM, 600 μM, and 700 μM) to triplicate wells containing 40 μl serum assay solution. For blank wells, use 40 μl dilution buffer.
  *The final [K*^+^*] in the wells will be 0 μM, 100 μM, 150 μM, 200 μM, 250 μM, 300 μM, and 350 μM*.
  *Be very careful with pipetting. GEPII 1.0 is highly sensitive for K*^+^ *and slight deviations or pipetting mistakes will drastically affect the calibration curve, leading to errors in calculating [K*^+^*] from the serum samples. Avoid air bubbles in pipetting, as they may alter fluorescence measurements. If air bubbles have been produced, try to destroy them using sterile needles*.
11. Measure samples using a CLARIOstar Plus fluorescence plate reader with the following settings. Be sure to remove the lid from the plate before taking measurements.
  Excitation: 430-20 nm
    Emission: 475-10 and 525-10 nm
    Dichroic filters: 455 and 480 nm
    Gain: 2,000 for both emissions with focal height adjusted to blank
    Top optics
12. Calculate the FRET ratio by determining the FRET/CFP fluorescence (525 nm/475 nm) of each well containing K^+^ calibration solution. Plot the FRET/CFP ratio versus [K^+^] as demonstrated in Figure 3A.
13. Calculate the FRET/CFP ratios of the blank wells, determine the average value, and then subtract that from each value of the calibration curve. Plot the blank-corrected FRET/CFP ratios (∆Ratio) against [K^+^] as demonstrated in Figure 3B, and fit the values using a one-phase decay. Create the equation of the curve and solve it for *x* as demonstrated in Figure 3C.
  *The formula is afterwards used to calculate the [K*^+^*] from the FRET/CFP ratio of GEPII 1.0*.

### Determine [K^+^] in serum samples

**Figure 4:**
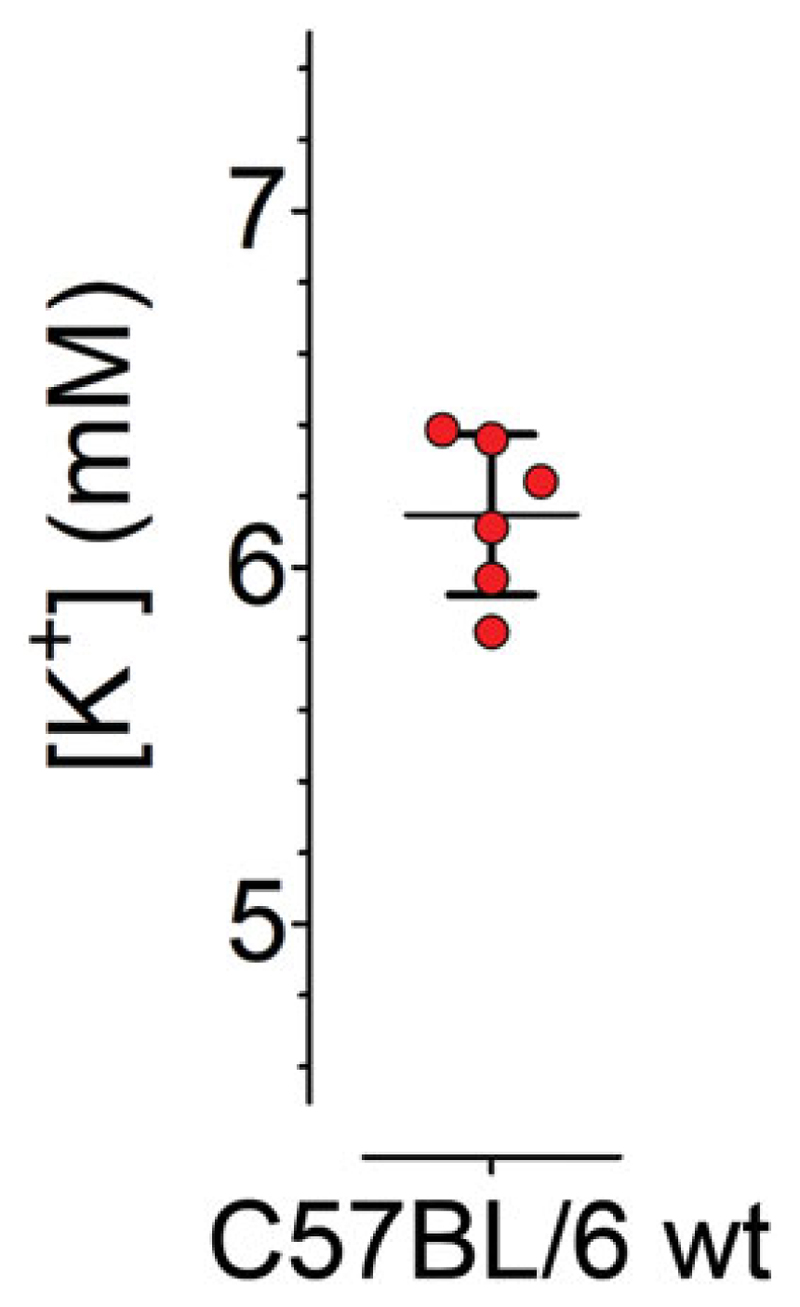
[K^+^] in serum samples of six C57BL/6 mice determined using GEPII 1.0. Each sample and the average ± SD are shown.

14. If serum samples were frozen, thaw them on ice.
15. Dilute 8 μl of each serum sample and K^+^ standard solution (5.0 and 8.0 mM) with 92 μl dilution buffer (1:12.5 dilution). Mix by inverting tubes. Do NOT vortex, as vortexing may lead to foaming.
  *The optimal dilution factor may vary depending on the expected [K*^+^*] within the samples. The final [K*^+^*] in the wells needs to fall within the range of the calibration curve. For murine serum, one can expect [K*^+^*] to be 5-8 mM. The initial dilution of 1:12.5 yields [K*^+^*] of 400-640 μM. A further 1:2 dilution with serum assay solution gives a final assay dilution of 1:25 (200-320 μM)*.
  *We recommend including at least two K*^+^ *standards as internal standards*.
16. For each [K^+^] determination, pipet 40 μl serum assay solution into duplicate wells of a 96-well plate. Include duplicate wells for the blank and both K^+^ standards.
  *We recommend performing all [K*^+^*] determinations at least in duplicate*.
17. Add 40 μl of each diluted sample and standard to the wells containing serum assay solution. Use dilution buffer for the blank wells.
  *Avoid air bubbles during pipetting, as air bubbles may alter the fluorescence measurements. If air bubbles have been produced, try to destroy them using sterile needles*.
18. Measure fluorescence as in step 11.
  *If you have generated a calibration curve earlier and did not use the indicated settings, do not change the measurement parameters! The samples and calibration curve must be measured with the same device settings*.
19. Calculate the FRET ratio by determining the FRET/CFP fluorescence (525 nm/475 nm) for each well.
20. Determine the average for the blank wells and subtract it from each sample and standard.
21. Verify the accuracy of the internal standards (K^+^ standard solutions) by calculating [K^+^] in mM using the formula generated from the calibration curve, as shown in Figure 3C.
  *Internal standards may vary* ±*5% of the target value. If K*^+^ *standard values deviate strongly, several parameters may have to be checked. See Critical Parameters and Troubleshooting for details*.
22. If the [K^+^] of the standards is within range, continue to calculate the [K^+^] for the samples. Determine the average of the duplicates for each sample and present data as desired.
  *Example data from sera of six animals are presented in Figure 4*.

## Basic Protocol 3: Measuring Extracellular [K^+^] with GEPII 1.0 for Visualization of Cell Viability over Time

The following protocol describes how to apply recombinant GEPII 1.0 for online visualization of cell viability over time by measuring extracellular [K^+^].

### Materials

Cells of interest and appropriate culture medium
  PBS (see recipe)
  Trypsin solution (see recipe)
  Cell wash buffer (see recipe)
  Cell assay solution (see recipe)
  850 μM digitonin stock solution (see recipe)

Humidified 37°C, 5% CO_2_ incubator
  30-ml polystyrene multipurpose container (Greiner Bio-One, cat. no. 201170)
  Refrigerated centrifuge (Sorvall RT6000B)
  Cell counting chamber (e.g., Bürker-Türk, VWR, cat. no. HECH40444702)
  96-well black polystyrene cell culture microplates, clear F-bottom (Greiner Bio-One, cat. no. 655090)
  Fluorescence plate reader (CLARIOstar Plus, BMG Labtech)

*NOTE:* All of the following steps should be performed in a sterile environment. All buffers and solutions used on cells should be prewarmed to 37°C.

### Prepare cells

**Figure 5:**
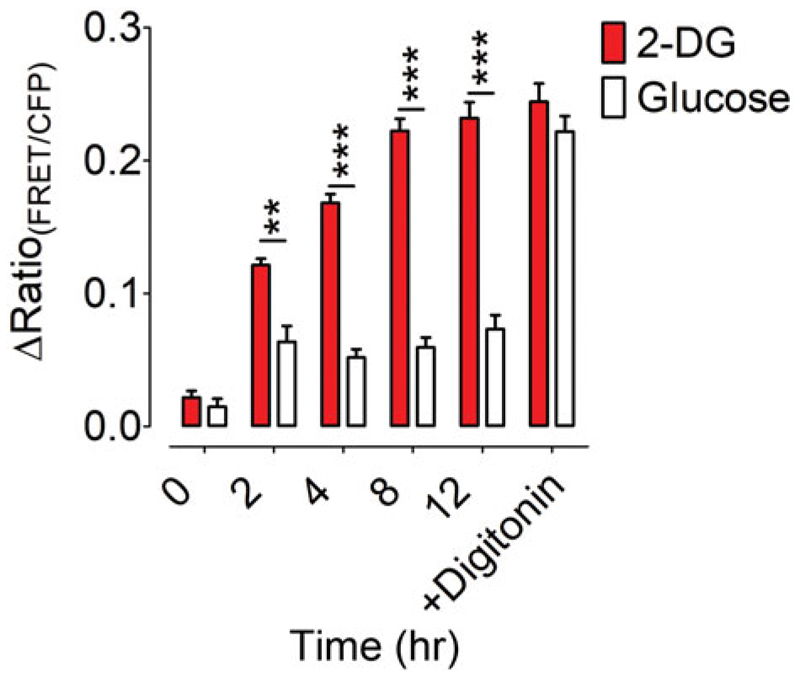
[K^+^] in supernatant of INS-1 832/13 cells at different times after cell treatment as determined using GEPII 1.0. Graph represents blank-corrected FRET ratio signals (∆Ratio_(FRET/CFP)_) after 0, 2, 4, 8, or 12 hr treatment with 10 mM glucose (white bars) or 10 mM 2-deoxyglucose (2-DG, red bars), or after application of 50 μM digitonin. *n* = 10 measurements for both conditions and each time point; **p < .005, ***p < .001, unpaired *t*-test.

1. Cultivate cells of interest to a confluency of ~80% using appropriate culture medium and a humidified incubator at 37°C with 5% CO_2_.
  *The initial number of cells needed depends on experimental need. Assuming one 10-cm dish at* ~*80% confluency has* ~*10 million cells, this is sufficient for* ~*200 wells (or two 96-well plates)*.
  *The sample data in Figure 5 were generated using INS-1 832/13 rat insulinoma cells (Merck Millipore, cat. no. SCC207) cultured in supplemented RPMI 1640 medium (see recipe)*.
2. One day before the experiment, wash cells with PBS and trypsinize them. For a 10-cm dish, use ~3 ml trypsin solution and incubate 2-4 min in a humidified 37°C incubator.
3. Suspend floating cells in 10 ml medium to stop trypsinization.
4. Transfer suspension to a 30-ml multipurpose container and centrifuge at 200 × *g* for 10 min (3,000 rpm in Sorvall RT6000B).
5. Remove supernatant and wash cells with 10 ml PBS, centrifuging again at 200 × *g* for 10 min.
6. Remove PBS, carefully resuspend cell pellet in 10 ml supplemented RPMI 1640, and place on ice to prevent cell adherence.
7. Determine cell density (number/ml) using a Bürker-Türk counting chamber or any other method of choice. Adjust to ~250,000 cells/ml.
8. Seed 200 μl suspension (~50,000 cells) in the desired number of wells of a black 96-well cell culture plate with a clear bottom. For each condition, include at least two wells containing 200 μl medium alone (no cells) for blank measurements.
9. Culture cells overnight, then check cell density. Proceed to the next step when cells are 70%-80% confluent.

### Perform assay

10. Wash wells twice with 250 μl cell wash buffer.
11. Remove cell wash buffer from all wells and replace with 80 μl cell assay solution containing either the compound of interest or vehicle. Use at least two blank wells per condition.
  CAUTION: *Be careful with pipetting. The more exact the volume is in each well, the better the results will be, as the [K*^+^*] in the supernatant is dependent on the volume of the supernatant*.
12. Measure CFP and FRET signals of GEPII 1.0 over time using a CLARIOstar Plus fluorescence plate reader with the following settings.
  Excitation: 430-20 nm
    Emissions: 475-10 and 525-10 nm
    Dichroic filters: 455 and 480 nm
    Gain: 2,000 for both emissions with focal height adjusted to blank
    Bottom optics
  CAUTION: *To prevent evaporation, do not remove the lid from the 96-well plate prior to the measurements*.
  *The temporal resolution of the measurement will depend on the cell treatment. The faster the cells are expected to undergo cell death, the higher the temporal resolution needed*.
13. At the end of measurement, add 5 μl of 850 μM digitonin stock solution to all wells including blanks (final 50 μM).
  *Application of digitonin will permeabilize all cell membranes and is important in order to ensure the same cell numbers under all conditions*.
14. Calculate the FRET ratio signal of GEPII 1.0 by determining the FRET/CFP fluorescence (525 nm/475 nm) for all wells including blanks.
15. Perform blank correction of FRET ratio signals from wells containing cells using the respective blanks. If several wells were used as a blank, calculate the average blank value and subtract it from each well of the respective condition.
16. Analyze and present the FRET ratio signal of GEPII 1.0 over time as demonstrated in Figure 5.

## Basic Protocol 4: Generation of a GEPII 1.0 Calibration Curve for Estimating the Number of Dead Cells

The following protocol describes how to quantify the number of dead cells using recombinant GEPII 1.0 for quantification of extracellular [K^+^]. Refer to Basic Protocol 3 for all materials needed.

### Prepare cells

**Figure 6:**
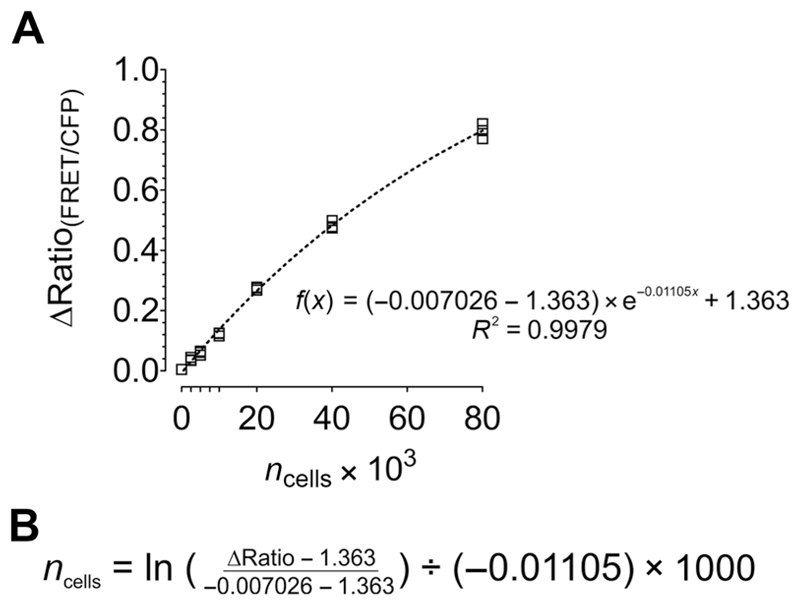
Generation of a calibration curve for calculating cell number from the FRET ratio signal of GEPII 1.0. (**A**) Increasing numbers of HeLa cells in DMEM were seeded into the wells of a 96-well plate. After cell permeabilization using 50 μM digitonin, the FRET ratio signal of GEPII 1.0 was recorded. Blank-corrected FRET ratio signal (∆Ratio_(FRET/CFP)_) is plotted against cell number. Values were fitted using a one-phase decay. The equation of the curve and *R^2^* value are indicated. *n* = 3 for each cell number. (**B**) Formula for calculating cell number from the ΔFRET ratio signal of GEPII 1.0 obtained by solving the equation demonstrated in (A).

1. Cultivate cells of interest to a confluency of ~80% using the appropriate culture medium and a humidified incubator at 37°C with 5% CO_2_.
  *It is critical to note that the determined regression curve and formula are cell-type specific and are not applicable for other cell lines than the one used to generate them. The sample data in Figure 6 were generated using HeLa cells cultured in DMEM (see recipe)*.
2. On the day of the experiment, wash cells with PBS and trypsinize them. For a 10-cm dish, use ~3 ml trypsin solution and incubate 2-4 min in a humidified 37°C incubator.
3. Suspend floating cells in 10 ml medium to stop trypsinization.
4. Transfer suspension to a 30-ml multipurpose container and centrifuge at 200 × *g* for 10 min (3,000 rpm in Sorvall RT6000B).
5. Remove supernatant and wash cells with 10 ml cell wash buffer, centrifuging again at 200 × *g* for 10 min.
6. Remove supernatant and carefully resuspend cells in 5 ml cell wash buffer.
7. Determine cell density (number/ml) using a Bürker-Türk counting chamber or any other method of choice.
  *Count cells as precisely as possible, as deviations in the assumed cell number will affect the estimated number of dead cells for Basic Protocol 3*.

### Perform assay

8. Dispense increasing cell numbers into the wells of a black 96-well cell culture plate with clear bottom and adjust the volume of each well to 40 μl using cell wash buffer. Include blank wells containing 40 μl cell wash buffer without cells.
  *We recommend performing at least triplicates for each cell number tested. Appropriate cell numbers range from 1,000 to 200,000, depending on cell type. For the example in Figure 6, we used 0, 2,500, 5,000, 10,000, 20,000, 40,000, and 80,000 HeLa cells*.
9. Add 35 μl cell assay solution to all wells.
10. Add 5 μl of 850 μM digitonin stock solution to all wells (final 50 μM) and incubate for 10 min for full permeabilization of all cells.
  *Application of digitonin will permeabilize all cell membranes and allow determination of the K*^+^ *released from defined cell numbers*.
11. Measure CFP and FRET signals of GEPII 1.0 over time using the CLARIOstar Plus fluorescence plate reader with the following settings:
  Excitation: 430-20 nm
    Emissions: 475-10 and 525-10 nm
    Dichroic filters: 455 and 480 nm
    Gain: 2,000 for both emissions with focal height adjusted to blank
    Bottom optics
  CAUTION: *To prevent evaporation of buffer, do not remove the lid from the 96-well plate prior to measurement*.
  *If you have measured cell viability over time earlier and did not use these settings, do not change the measurement parameters! The samples and calibration curve must be measured with the same device settings*.
12. Calculate the FRET ratio signal of GEPII 1.0 by determining the FRET/CFP fluorescence (525 nm/475 nm) for all wells including blanks.
13. Perform blank correction of FRET ratio signals from wells containing cells using the respective blanks. If several wells were used as a blank, calculate the average blank value and subtract it from each well of the respective condition.
14. Analyze and present the FRET ratio signal of GEPII 1.0 as demonstrated in Figure 6A.
15. Fit values using a proper regression, create the equation of the curve, and solve for *x* as demonstrated in Figure 6B.
  *The formula allows calculation of cell number from the [K*^+^*] and can be applied to data obtained in Basic Protocol 3*.

## Reagents and Solutions

### Cell assay solution

Cell wash buffer (see recipe)
  400 nM GEPII 1.0 (see Basic Protocol 1)
  Desired test compound or vehicle
  Sterilize using 0.2-μm sterile filters (Sarstedt, cat. no. 83.1826.001)
  Prepare fresh daily
  Keep on ice in the dark
  *Assay solution should always be prepared with test compound and with vehicle to provide appropriate blanks for the assay*.
  CAUTION: *Do not apply too much pressure during filtration, as this may degrade recombinant GEPII 1.0*.

### Cell wash buffer

143 mM ultrapure NaCl (Carl Roth, cat. no. 5741.2)
  10 mM HEPES (Carl Roth, cat. no. 9105.3)
  2 mM CaCl_2_ (Carl Roth, cat. no. CN93.2)
  1 mM MgCl_2_ (Carl Roth, cat. no. KK36.2)
  Adjust pH to 7.3 using HCl (Carl Roth, cat. no. 9787.1) or *N*-methyl-d-glucamine (Sigma-Aldrich, cat. no. M2004)
  Sterilize using 0.2-μm sterile filters (Sarstedt, cat. no. 83.1826.001)
  Store up to 2 weeks at 4°C

### Column storage buffer

0.02% NaN_3_ (Sigma-Aldrich, S2002-25G) in distilled water
  Store up to 6 months at 4°C

### Digitonin stock solution, 850 μM

Dissolve digitonin (Sigma-Aldrich, cat. no. D5628-1G) in DMSO at a concentration of 850 μM. Prepare fresh before use and store at room temperature for up to 12 hr.

### Dilution buffer

10 mM HEPES (Carl Roth, cat. no. 9105.3)
  0.05% (v/v) Triton X-100 (Carl Roth, cat. no. 3051.3)
  Adjust pH to 7.3 using HCl (Carl Roth, cat. no. 9787.1) or *N*-methyl-d-glucamine (Sigma-Aldrich, cat. no. M2004)
  Store up to 2 weeks at 4°C

### DMEM

For 1 liter:
  8.3 g Dulbecco’s Modified Eagle’s Medium (Sigma-Aldrich, cat.no. D5030-10L)
  1g D-(+)-glucose monohydrate (Carl Roth, cat.no. 6887.1)
  0.584 g l-glutamine (Sigma-Aldrich, cat.no. G3126-100G)
  3.7 g NaHCO_3_ (Carl Roth, cat.no. HN01.1)
  6 g HEPES (Carl Roth, cat.no. 9105.3)
  Adjust pH first to 7.9 using 5 N NaOH (Carl Roth, cat.no. 6771.3)
  Adjust pH to 7.4 using CO_2_
  Add 10 ml penicillin-streptomycin (Thermo Fisher Scientific, cat.no. 15140122)
  5 ml amphotericin B (Thermo Fisher Scientific, cat.no. 15290026)
  Filter sterilize
  Store at 4°C (stable at least 3 months)
  Before use, add 10% (v/v) sterile FBS (Thermo Fisher Scientific, cat.no. 10270-106)

### Glycerol solution, 50% (v/v)

Dilute an equal amount of glycerol (Carl Roth, cat. no. 6967.1) in distilled water and autoclave. Store at room temperature (stable at least 1 year if kept sterile).

### High-salt purification buffer

100 mM Na_2_HPO_4_ (Carl Roth, cat. no. T876.1)
  200 mM NaCl (Carl Roth, cat. no. P029.2)
  200 mM imidazole (Carl Roth, cat. no. X998.2)
  Adjust pH to 8.0 using HCl (Carl Roth, cat. no. 9787.1) or NaOH (Carl Roth, cat. no. 6771.3)
  Store up to 2 weeks at room temperature

### K^+^ calibration solutions

10 mM HEPES (Carl Roth, cat. no. 9105.3)
  0.05% (v/v) Triton X-100 (Carl Roth, cat. no. 3051.3)
  KCl (Carl Roth, 5346.1) at concentrations of 60 μM, 200 μM, 300 μM, 400 μM, 500 μM, 600 μM, 700 μM, 2 mM, 6 mM, and 20 mM
  Adjust pH to 7.3 using HCl (Carl Roth, cat. no. 9787.1) or *N*-methyl-d-glucamine (Sigma-Aldrich, cat. no. M2004)
  Prepare fresh before use

### K^+^ standard solutions

10 mM HEPES (Carl Roth, cat. no. 9105.3)
  0.05% (v/v) Triton-X 100 (Carl Roth, cat. no. 3051.3)
  5.0 and 8.0 mM KCl (Carl Roth, cat. no. 5346.1)
  Adjust pH to 7.3 using HCl (Carl Roth, cat. no. 9787.1) or *N*-methyl-d-glucamine (Sigma-Aldrich, cat. no. M2004)
  Prepare fresh before use

### Luria-Bertani (LB) medium

10 g/L NaCl (Carl Roth, cat. no. P029.2)
  10 g/L tryptone/peptone ex casein (Carl Roth, cat. no. 8952.1)
  5 g/L yeast extract (Carl Roth, cat. no. 2363.2)
  Autoclave
  Store at room temperature (stable at least 6 months)

### Lysis buffer

100 mM Na_2_HPO_4_ (Carl Roth, cat. no. T876.1)
  200 mM NaCl (Carl Roth, cat. no. P029.2)
  10 mM imidazole (Carl Roth, cat. no. X998.2)
  Adjust pH to 8.0 using HCl (Carl Roth, cat. no. 9787.1) or NaOH (Carl Roth, cat. no. 6771.3)
  Store up to 1 month at 4°C

### Phosphate-buffered saline (PBS)

For 1 liter:
  8 g NaCl (Carl Roth, cat. no. P029.2)
  0.2 g KCl (Carl Roth, cat. no. 5346.1)
  1.44 g Na_2_HPO_4_ (Carl Roth, cat. no. T876.1)
  0.24 KH_2_PO_4_ (Carl Roth, cat. no. 3904.1)
  Adjust pH to 7.4 using HCl (Carl Roth, cat. no. 9787.1)
  Autoclave
  Store at 4°C (stable at least 6 months)

### SEC buffer

10 mM HEPES (Carl Roth, cat. no. 9105.3)
  Adjust pH to 7.3 using HCl (Carl Roth, cat. no. 9787.1) or *N*-methyl-d-glucamine (Sigma Aldrich, cat. no. M2004)
  Store up to 2 weeks at 4°C

### Serum assay solution

Dilution buffer (see recipe)
  400 nM GEPII 1.0 (see Basic Protocol 1)
  Prepare fresh daily
  Keep on ice in the dark

### SOC medium

Prepare in 100 ml distilled water:
0.5 g yeast extract (Carl Roth, cat. no. 2363.2)
  2 g tryptone/peptone ex casein (Carl Roth, cat. no. 8952.1)
  58 mg NaCl (Carl Roth, cat. no. P029.2)
  20 mg KCl (Carl Roth, cat. no. 5346.1)
  100 mg MgCl_2_ (Carl Roth, cat. no. KK36.1)
  120 mg MgSO_4_ (Carl Roth, cat. no. 0682.1)
  Autoclave
  Add 400 mg d-(+)-glucose monohydrate (Carl Roth, cat. no. 6887.1)
  Sterilize using a 0.2-μm sterile filter (Sarstedt, cat. no. 83.1826.001)
  Prepare 1-ml aliquots in 1.5-ml microcentrifuge tubes
  Store at −80°C (stable for several years)

### Supplemented RPMI 1640 medium

Gibco RPMI 1640 medium (Thermo Fisher Scientific, cat. no. 12633012)
  10% (v/v) FBS (Thermo Fisher Scientific, cat. no. 10270-106)
  10 mM HEPES (Carl Roth, cat. no. 9105.3)
  1 mM sodium pyruvate (Thermo Fisher Scientific, cat. no. 11360-039)
  0.05 mM 2-mercaptoethanol (Carl Roth, cat. no. 4227.3)
  1% (v/v) penicillin-streptomycin (10,000 U/ml; Thermo Fisher Scientific, cat. no. 15140122)
  1% (v/v) amphotericin B (Thermo Fisher Scientific, cat. no. 15290026)
  Filter sterilize
  Store at 4°C (stable at least 3 months)

### Trypsin solution

1 L PBS (see recipe)
  500 mg trypsin (Sigma-Aldrich, cat. no. T7409-10G)
  200 mg EDTA (Carl Roth, CN06.1)
  Filter sterilize
  Store 25-ml aliquots at −20°C (stable at least 1 year)

### Wash buffer 1

100 mM Na_2_HPO_4_ (Carl Roth, cat. no. T876.1)
  200 mM NaCl (Carl Roth, cat. no. P029.2)
  40 mM imidazole (Carl Roth, cat. no. X998.2)
  Adjust pH to 8.0 using HCl (Carl Roth, cat. no. 9787.1) or NaOH (Carl Roth, cat. no. 6771.3)
  Store up to 2 weeks at room temperature

### Wash buffer 2

100 mM Na_2_HPO_4_ (Carl Roth, cat. no. T876.1)
  1 M NaCl (Carl Roth, cat. no. P029.2)
  10 mM imidazole (Carl Roth, cat. no. X998.2)
  Adjust pH to 8.0 using HCl (Carl Roth, cat. no. 9787.1) or NaOH (Carl Roth, cat. no. 6771.3)
  Store up to 2 weeks at room temperature

## Commentary

### Background Information

The first successful design of a FRET-based probe based on a yellow and cyan fluorescent protein (FP) variant was reported in 1997 by Miyawaki and colleagues, who introduced a Ca^2+^-sensitive FRET-based sensor (Miyawaki et al., 1997). Today, a wide variety of these FRET-based indicators is available, including sensors for various metal ions, pH, cell metabolites, or even small molecules, often having short half-lives (Bischof et al., 2019; Burgstaller et al., 2019; Imamura et al., 2009; Tanimura, 2011). All of these probes are based on diverse FP variants, enabling a FRET-based read-out, fused to a specific analyte-binding domain, undergoing a conformational rearrangement upon analyte binding. In 2016, Ashraf et al. unraveled the function of the bacterial protein Kbp, formerly known as YgaU (Ashraf et al., 2016). They demonstrated that Kbp represents a K^+^-binding protein in *E. coli* that is important to ensure normal growth of the bacteria under conditions of high extracellular [K^+^]. Upon K^+^ binding, Kbp undergoes a huge conformational rearrangement, from an elongated conformation towards a spherical one (Ashraf et al., 2016).

Based on this protein, we recently developed a series of genetically encoded K^+^ indicators, the GEPIIs (Bischof et al., 2017). These GEPIIs consist of a cyan and a yellow fluorescent protein, namely monomeric super enhanced CFP (mseCFP) and circularly permuted Venus (cpV), a well-characterized FP FRET pair, fused to the N and C terminus of Kbp, respectively. In the absence of K^+^, FRET efficiency is low, yielding high donor fluorescence. However, upon K^+^ binding to the construct, the protein undergoes a conformational rearrangement, leading to increased FRET and decreased cyan emission (Bajar,Wang, Zhang, Lin & Chu, 2016). While several mutated GEPII variants showed K^+^ affinities suitable for intracellular K^+^ measurements, the GEPII variant referred to as GEPII 1.0, containing the wild-type Kbp, showed a very high affinity and specificity for K^+^. Based on this high sensitivity, we hypothesized that the recombinant GEPII 1.0 protein represents a valuable tool for quantification of extracellular [K^+^] in various biological samples. Our data emphasized that GEPII 1.0 is able to determine [K^+^] in serum and urine samples of healthy and diseased human donors as precisely as the gold-standard method for [K^+^] measurements, the ISE. The use of GEPII 1.0 for quantification of [K^+^] in body fluids requires only a fraction of the sample volume required for determination by ISE. Thus, we exploited this high sensitivity and accuracy for quantification of [K^+^] in murine serum, urine, and even bile samples, as mice possess very limited amounts of these biological fluids. Using GEPII 1.0 for these measurements will in future allow repetitive sample collection from one given animal over time, without need for its sacrifice. In this work, we provide scientists a detailed step-by-step manual for purifying the recombinant GEPII 1.0 protein, preparing murine serum samples, and quantifying serum [K^+^] using GEPII 1.0.

Furthermore, our recent data demonstrated the suitability of GEPII 1.0 for online visualization of cell viability over time by measuring extracellular [K^+^]. As vital cells maintain a steep K^+^ gradient towards the plasma membrane, the measurement of extracellular [K^+^] represents a facile method to visualize cell viability with high temporal resolution, without the need of expensive chemicals, which often allow only end-point measurements. We also demonstrate an example for estimating the number of dead cells using this K^+^-sensitive, FRET-based probe.

To our knowledge this is the first time that a genetically encoded, FRET-based biosensor has been applied as a recombinant, purified protein for the quantification of an analyte within biological samples.

### Critical Parameters and Troubleshooting

One of the most critical parameters of these protocols is the purification of recombinant GEPII 1.0 from *E. coli*. It is essential to test the functionality of recombinant GEPII 1.0 after purification to demonstrate and ensure the functionality of the probe for reporting and responding in a concentration-dependent manner to increasing [K^+^].

It is of utmost importance to use high-quality distilled water and ultrapure graded salts when eluting the protein from the size-exclusion column, as pre-saturation of the recombinant probe will drastically affect the dynamic range of the sensor. To quantitate and identify a possible pre-saturation with K^+^, one may include wells containing K^+^ chelators such as poly(sodium 4-styrenesulfonate) (Sigma-Aldrich, cat. no. 434574-100G). Typically, 50 μMconcentrations of a given chelator are capable of buffering at least 20-30 mM of K^+^. In cases of a drastically reduced GEPII 1.0 FRET ratio signal in wells containing the chelator, GEPII 1.0 is pre-saturated with K^+^.

The primary reason for K^+^ impurity of the recombinant protein solution might be the use of contaminated SEC buffer or impurities on the size-exclusion column itself. Under such circumstances, one can restart the protein purification, increase the buffer volume in the wash step prior to size exclusion, and control the recipe and chemicals used for preparing the SEC buffer. Another possibility is to perform desalting protocols after size-exclusion chromatography.

The exact determination of [K^+^] within biological samples using this GEPII 1.0 from bacterial expression requires the generation of a K^+^ calibration curve. Importantly, the calibration solutions and protein-containing solutions must be prepared as precisely as possible. Improper preparation of the calibration curve will cause incorrect calculations of [K^+^] from all samples. Determining the [K^+^] of diverse K^+^ standard solutions can assist in the identification of pipetting, dilution, or calculation errors.

As the K^+^ sensitivity of GEPII 1.0 appears temperature dependent in vitro, [K^+^] measurements need to be performed at constant temperature settings of the fluorescence plate reader. In principle, theK^+^ sensitivity decreases with increasing temperature and vice versa; thus, measuring at room temperature or below leads to lowered detection limits of GEPII 1.0 for K^+^.

For additional troubleshooting, see Table 1.

### Understanding Results

Mammalian organisms tightly control their extracellular [K^+^] to ensure proper function of all cell types and organs (Palmer, 2015). Alterations of the extracellular [K^+^] are associated with severe pathological alterations and mostly require urgent medical treatment. Frequently, renal dysfunction and insufficiencies are associated with increased serum K^+^ levels (Kunis & Charney, 1981), and thus the measurement of serum [K^+^] often serves as an indication for renal disorders. Using GEPII 1.0, we have demonstrated drastic differences in the extracellular [K^+^] of healthy control mice and mice suffering surgically inflicted ischemia reperfusion injury. Our data emphasize that the measurement of [K^+^] within serum samples of small laboratory animals in various pathological models can deepen our understanding of the organismal K^+^ homeostasis. Furthermore, considering the small sample volume required for determination of [K^+^] using GEPII 1.0, scientists can follow [K^+^] within serum upon drug application of one given animal over time, which might be of essential importance for drug development and verification.

Because vital cells maintain a steepK^+^ gradient across the plasma membrane, GEPII 1.0 can further be used for visualizing cell viability over time (Bischof et al., 2017; Palmer, 2015). Cells undergoing cell death release K^+^ into their extracellular environment, which can be measured using GEPII 1.0. The use of the recombinant protein in cell supernatants thereby allows online visualization of cell death over time in the presence of different compounds or their respective vehicle controls, which is not easily feasible using standard cell viability assays that represent end-point measurements rather than online assays. Additionally, the signal received from different cells can be calibrated by using defined cell numbers, allowing a calculation of real cell numbers that underwent cell death.

### Time Considerations

Basic Protocol 1 takes a total of ~29 hr. This includes an overnight incubation (~12-hr) for bacterial transformation with GEPII 1.0, inoculation of LB medium and induction of protein expression (~8 hr), protein purification from the cell lysate (~8 hr), and functionality testing of the purified GEPII 1.0 (~1 hr).

Basic Protocol 2 takes ~2 hr, including collection of murine blood samples and preparation of serum (~30 min), generation of the K^+^ calibration curve for GEPII 1.0 (~30 min), and determination of [K^+^] in murine serum samples (~1 hr).

Basic Protocol 3 takes at least 26.5 hr. Trypsinization, counting, and seeding of cells in 96-well plates takes ~1.5 hr. Cultivation of cells in 96-well plates takes ~24 hr, and preparation of the cells for the cell viability assay takes ~1 hr. The duration of the experiment depends on the research question.

Basic Protocol 4 takes ~2 hr, including cell trypsinization and counting (~1 hr) and cell seeding, permeabilization, and determination of [K^+^] using GEPII 1.0 (~1 hr).

## Figures and Tables

**Table 1:** Troubleshooting

Problem	Solution
Bacterial culture does not grow after plasmid transformation	Check transformation protocol, especially the temperature and time used for heat shock
	Check formulation of LB medium
	Check for proper antibiotics
Bacteria are not greenish to yellow in color after induction of protein expression	Check bacterial strain used for transformation of GEPII 1.0
	Check protein expression by SDS-PAGE
	Check transformation efficiency of GEPII 1.0 into bacteria
	Check concentration of IPTG used to induce protein expression
	Check bacteria for inclusion bodies. Reduction of IPTG and reduced incubation temperature after induction of expression may slow protein folding and prevent inclusion body formation.
No protein or very low protein concentration after purification	Check all buffers for correct formulation
	Check for proteolytic cleavage of the protein; eventually add protease inhibitors to cell lysate
	Check devices, substances, and equipment for proper storage and function
	Check concentrator tubes for correct cut-off
GEPII 1.0 does not respond to increasing [K^+^]	Check for possible K^+^ pre-saturation by application of K^+^ chelators
	Check protein concentration used for [K^+^] measurements
	Check that protein is full length by SDS-PAGE
	Check settings of fluorescence plate reader
Serum samples appear hemolytic	Check centrifugation speed for blood samples
[K^+^] determination of K^+^ standard solutions is wrong	Check K^+^ calibration and standard solutions for correct formulation
	Check calculation formula for determining [K^+^]
	Check settings of fluorescence plate reader
No change in [K^+^] is reported in cell viability assays	Check GEPII 1.0 for functionality
	Check all buffers and solutions for correct formulation
	Check settings of fluorescence plate reader
	Check cell numbers seeded in 96-well plates
	Check for bacterial contamination
No change in [K^+^] reported in cell viability assays upon application of digitonin	Check GEPII 1.0 for functionality
	Check all buffers and solutions for correct formulation
	Check settings of fluorescence plate reader
	Check cell number seeded in 96-well plates
	Check for bacterial contamination
	Check concentration of digitonin used to permeabilize cells
[K^+^] released from permeabilized cells does not correlate with cell number	Check GEPII 1.0 for functionality
	Check all buffers and solutions for correct formulation
	Check settings of fluorescence plate reader
	Check for bacterial contamination
	Check cell numbers used
